# From conventional to self-ligating bracket systems: Is it possible to aggregate the experience with the former to the use of the latter?

**DOI:** 10.1590/2176-9451.19.3.139-157.sar

**Published:** 2014

**Authors:** Anderson Capistrano, Aldir Cordeiro, Danilo Furquim Siqueira, Leopoldino Capelozza Filho, Mauricio de Almeida Cardoso, Renata Rodrigues de Almeida-Pedrin

**Affiliations:** 1 Professor of Occlusion and Orthodontics, School of Dentistry of Recife (FOR-PE).; 2 Masters student in Orthodontics, Sacred Heart University (USC).; 3 Professor, Department of Orthodontics, Undergraduate and Postgraduate Program, USC.

**Keywords:** Orthodontic brackets, Angle Class III malocclusion, Facial pattern

## Abstract

**Introduction:**

Orthodontics, just as any other science, has undergone advances in technology that aim at improving treatment efficacy with a view to reducing treatment time, providing patients with comfort, and achieving the expected, yet hardly attained long-term stability. The current advances in orthodontic technology seem to represent a period of transition between conventional brackets (with elastic ligatures) and self-ligating brackets systems. Scientific evidence does not always confirm the clear clinical advantages of the self-ligating system, particularly with regard to reduced time required for alignment and leveling (a relatively simple protocol), greater comfort for patients, and higher chances of performing treatment without extractions - even though the number of extractions is more closely related to patient's facial morphological pattern, regardless of the technique of choice. Orthodontics has recently and brilliantly used bracket individualization in compensatory treatment with a view to improving treatment efficacy with lower biological costs and reduced treatment time.

**Objective:**

This paper aims at presenting a well-defined protocol employed to produce a better treatment performance during this period of technological transition. It explores the advantages of each system, particularly with regards to reduced treatment time and increased compensatory tooth movement in adult patients. It particularly addresses compensable Class III malocclusions, comparing the system of self-ligating brackets, with which greater expansive and protrusive tooth movement (maxillary arch) is expected, with conventional brackets Capelozza Prescription III, with which maintaining the original form of the arch (mandibular arch) with as little changes as possible is key to yield the desired results.

## Abstract (pt)

**Introdução:**

a Ortodontia passa, como toda ciência, por constantes evoluções tecnológicas que buscam aumentar a efetividade da abordagem terapêutica, visando a diminuição do tempo de tratamento, o aumento do conforto para os pacientes, bem como a obtenção da tão almejada, e pouco alcançada, estabilidade em longo prazo. O estágio atual de desenvolvimento tecnológico da Ortodontia representa, ao que tudo indica, uma fase de transição entre os sistemas convencionais de ligação (com módulos elásticos) e os chamados autoligáveis. As evidências científicas nem sempre consubstanciam a clara percepção clínica das vantagens desse sistema, no que diz respeito a um menor tempo de alinhamento e nivelamento, uma relativa simplificação técnica, maior conforto para os pacientes, além do aumento da capacidade de tratamento sem extrações - embora essa indicação esteja mais ligada à avaliação do padrão morfológico facial, e menos a qualquer escolha técnica. Desde um passado recente e não menos brilhante, a Ortodontia vem utilizando a individualização de braquetes para tratamentos compensatórios, buscando aumentar a efetividade da abordagem terapêutica, com menores custos biológicos e menor tempo de tratamento.

**Objetivo:**

o presente artigo tem como objetivo apresentar um protocolo bem definido de melhor aproveitamento dessa fase de transição tecnológica, buscando explorar o que cada sistema tem de melhor, principalmente sob a óptica da redução do tempo de tratamento e aumento da capacidade de movimentação dentária compensatória em pacientes adultos. Especificamente, serão abordadas as más oclusões de Classe III compensáveis, usando o sistema de braquetes autoligáveis onde se deseja maior capacidade de movimento expansivo e protrusivo (arcada superior) e braquetes convencionais Prescrição III Capelozza^®^ onde a manutenção da forma com mínima mudança (arcada inferior) é imprescindível para a obtenção dos resultados almejados.

## INTRODUCTION

Patients with Class III facial pattern and severe Angle Class III malocclusion pose difficulties for the clinical management of sagittal relationship between maxilla and mandible. Should surgery not be an option, clinical management is mainly concerned about guiding the mechanics, since its onset, in order to produce effects that meet the compensatory characteristics of the pattern. In the mandibular arch: restricted buccal tipping of incisors; maintenance of reduced mesial angulation of anterior lower teeth (except for canines that are usually distally angulated and, now, will be uprighted); and, in the transversal plane, respect to mandatory dentoalveolar compensatory mechanism - a *sine qua non* condition for transverse adjustment between the arches. As for the therapeutic management of the maxillary arch, it highly welcomes transverse gains, increased mesial angulation of canines and controlled protrusion.

Analysis of Class III dental arches reveals that compensatory changes must be proportional to the degree of malocclusion. As these patients nearly always undergo a functional routine, at least temporarily, the exception will be if these compensatory changes do not occur.^1^

The essence of compensatory treatment performed with these patients is to adapt the concept of normality for the occlusal relationship which is strongly influenced by the degree of sagittal discrepancy between the arches. In these cases, the therapeutic goals are completely individualized and treatment protocol must respect the adapted concept of normality for the occlusal relationship. At compensatory treatment completion, maxillary incisors will be more protruded and buccally tipped in accordance with esthetic limitations; the maxillary arch will be more expanded or with a decreased lingual inclination of posterior teeth; and all upper teeth will be more mesially angulated. All these goals are set for the maxillary arch with a view to increasing its circumference and length. Conversely, opposite goals are set for the mandibular arch: mandibular incisors as well as posterior teeth more lingually tipped, with decreased mesial angulation for all other teeth.^2^

Orthodontics has continuously sought to improve the efficiency of treatment in the attempt to reduce its duration and chair time. Although average treatment lasts between 1 and 2 years, there is an ongoing attempt to reduce it. To this end, several techniques and appliances - including surgical procedures, vibratory stimulation, greater use of individualized archwires and brackets, as well as less frequent indications for tooth extraction - will still be recommended. This article explores three important aspects of such continuous progression: bracket individualization, self-ligating systems and mechanical customization used to achieve greater therapeutic efficacy.^3^

## WHEN TO TREAT?

Despite not being the primary objective of this article, it is worth noting that the compensatory approach of skeletal Class III patients must safely begin, at least theoretically, in patients whose mandibular growth has ceased. Patient must present signs of skeletal maturity - for girls, 24 months after menarche; whereas for boys, there must be signs of full pubescence, such as voice alterations and facial hair. Such signs may be confirmed by carpal radiograph which reveals that the patient has achieved Haag &Taranger's^4^ stage IJ - an indication that compensatory orthodontic treatment may begin or that there is a need for corrected treatment by means of orthognathic surgery. Unlike compensatory treatment of skeletal Class II malocclusions, should orthodontic treatment be performed before the patient achieves the stage of skeletal maturity, treatment stability is not guaranteed even if satisfactory occlusal correction is achieved.^5^

## CHOOSING BRACKETS AND LIGATION SYSTEMS IN EACH ONE OF THE DENTAL ARCHES

In order to facilitate one's understanding of the treatment protocol presented in this article, it is important to divide the choice of brackets and ligation system in accordance with each dental arch.

### Maxillary arch

Over the last years, self-ligating brackets have been given great emphasis, partially due to producing lower friction. The possibility of theoretically applying force of appropriate magnitude increases the chances of periodontal tissues producing a more physiological response, thus producing more effective dental movements and, as a result, decreasing side effects and reducing treatment time.^6,7^

Increase in treatment efficacy is defined as the achievement of results which are as good as or better than those obtained by conventional treatment, especially within a shorter period of time. Additionally, increased productivity brings along major benefits for both clinician and patient. In orthodontic treatment, these benefits include a reduced number of visits, reduced chair time, more comfortable treatment, clinical procedures that can be easily performed by the orthodontist, a decreased need for extractions, less invasive treatment procedures and minimized feelings of pain and anxiety for the patient. Additionally, other factors associated with treatment conclusion could also be included, namely: less decalcification or root resorption or even better occlusal outcomes. The major gains of self-ligating systems, which lay the groundwork for approaches that opt for this type of treatment, are as follows: safe and complete positioning of the arch into the slot of the self-ligating bracket, which allows greater control of tooth movement; less resistance to sliding between the bracket and the arch, which increases the expansive capacity of the system; quicker arch removal and placement with a consequent reduction in chair time.^8^

Transverse expansion produced by self-ligating systems is explained by low friction between the bracket and the leveling arch. This fact was demonstrated by a study conducted with 20 patients in which the authors used non-conventional low friction elastometers. Their results revealed significant transverse expansion during alignment and leveling without further protrusion.^9^

On the other hand, another research assessed patients treated with passive, active and conventional brackets and found no significant differences for the distance between canines, premolars and molars. It is worth noting that no statistically significant difference was observed in the three groups assessed. Furthermore, in the group treated with passive self-ligating brackets, the distance between canines as well as first and second premolars had slightly higher values in comparison to the other groups.^10^

A significant advantage of a good self-ligating system is its ability to produce higher friction in clinical situations that require movement of a tooth, or a group of teeth, to be restricted along the leveling arch. To this end, a conventional elastometer may be used.^11^

The influence of therapeutic goals over the mechanical management of self-ligating systems is strengthened by a convenient method that includes the use of stops. They are little extensions of telescopic tubes or U-shape 2 to 3-mm open hooks normally positioned in the midline with the primary objective of avoiding distal sliding of wire, which would invariably injure the patient. In the context of the treatment protocol presented in this paper, it is recommended that the stops be placed in the mesial surface of maxillary first molars with a view to favoring incisors protrusion and canine mesialization.^12 ^The possibility of fully exploring this capacity of producing expansion and protrusion within a shorter period of time and in a more effective and, perhaps, more biological manner is what explains our choice of using a self-ligating system to treat the maxillary arch.

### Mandibular arch

Individualized brackets were reintroduced and spread in Brazilian literature by Capelozza Filho et al.^13 ^This type of bracket created an irrevocable culture of customization in Orthodontics which aims at fully respecting the morphology of patient's original malocclusion and, as a result, setting individual therapeutic goals. Capelozza^®^ Prescription III brackets require considerably limited angulation (which certainly is the most important factor for customization), with zero degree for canines and incisors and increased lingual incisor torque (-6°). For this reason, they are an excellent treatment option to maintain or increase (in a controlled manner) the compensatory features naturally present at the mandibular arch in Class III. This set of brackets aims at minimizing protrusion and eliminating retroclination, which is key to achieve success of compensatory treatment conducted with this type of patient. Nevertheless, customization is clearly not restricted to the choice of brackets. It includes careful bonding, proper selection of more restricted diagrams for the mandibular arch, properly fitted wires and Class III elastic mechanics, all of which decisively participate in preserving what deserves to be kept and highlighting what should be increased.^2,5,13^ Particularly with regards to diagram, it seems important to consider that it is determined in an objective manner, that is, respecting the essence of the arch which, in Class III patients, tends to present an increase in the distance between canines and a decrease in the posterior width of the mandibular arch.^16^

According to the literature, mandibular canines of Class III patients present an average difference in angulation of approximately 5 degrees in a distal direction, in comparison to Class I patients. For this reason, Class III patients tend to promote a natural compensation of mandibular incisors. Conversely, their maxillary canines present a smaller difference in mesial angulation of 2 degrees. In short, Class III patients have less tipped mandibular canines (-1.75º) in comparison to Class I patients (3.5º). These values are very close to those suggested for compensation brackets (Prescription III^®^): zero degree for mandibular canines.^14^

There is a tendency towards lingual inclination of mandibular incisors in cases of naturally compensated Class III malocclusion, since incisors inclination tends to promote a movement of opposite direction and which is compensatory to the maladjustment that results from a maxillomandibular skeletal imbalance. In other words, Pattern III, Class III patients have maxillary incisors more buccally tipped and mandibular incisors with increased lingual inclination.^14^

It is difficult to preserve the natural compensatory characteristics of the mandibular arch with the use of self-ligating brackets because, if we compare the degree of expansion achieved by self-ligating and conventional systems, it is clear that there is a stronger tendency for the former to increase the width of the arch.^15 ^Therefore, since this effect does not agree with treatment primary objective - which consists of preserving the transverse dimension of the mandibular arch - the treatment protocol reported herein chose to use the system of conventional brackets.

## CASE REPORT 1

**Figure 1:**
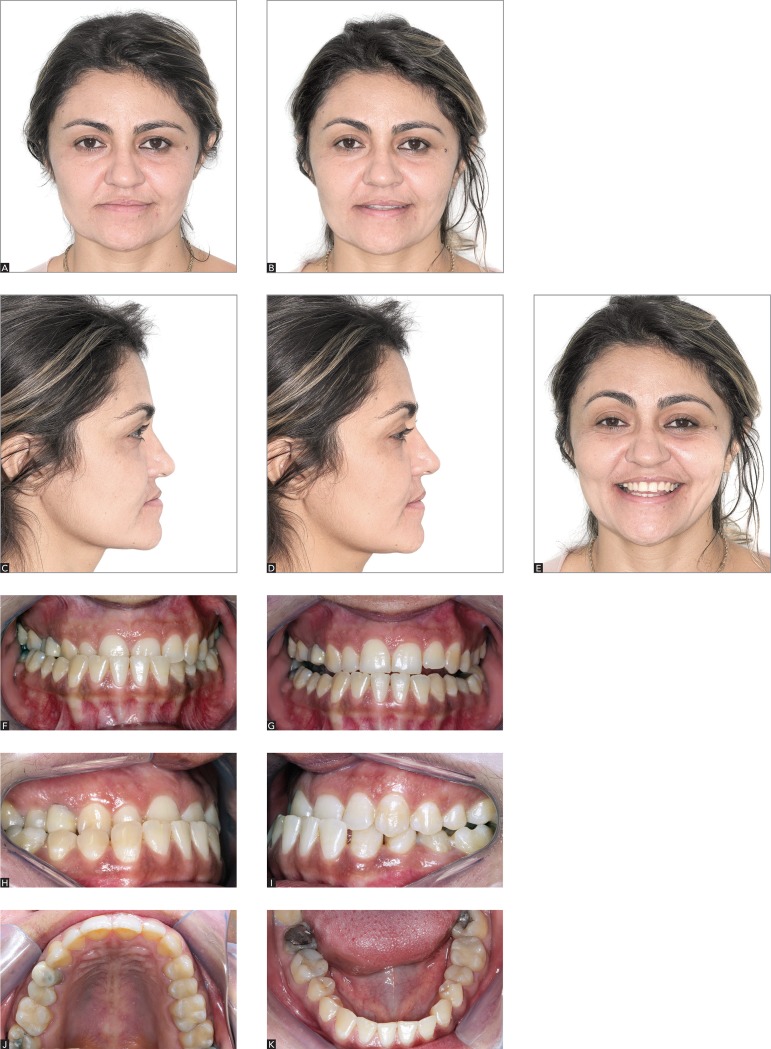
Initial photographs: **A**) frontal facial view in maximal intercuspation (MI); **B**) frontal facial view in centric relation (CR); **C**) facial profile in MI; **D**) facial profile in CR; **E**) smiling; **F**) intraoral frontal view in MI; **G**) intraoral frontal view in CR; **H**) intraoral lateral right view in MI. **I**) intraoral lateral left view in MI; **J**) intraoral occlusal maxillary view; **K**) intraoral occlusal mandibular view.

**Figure 2:**
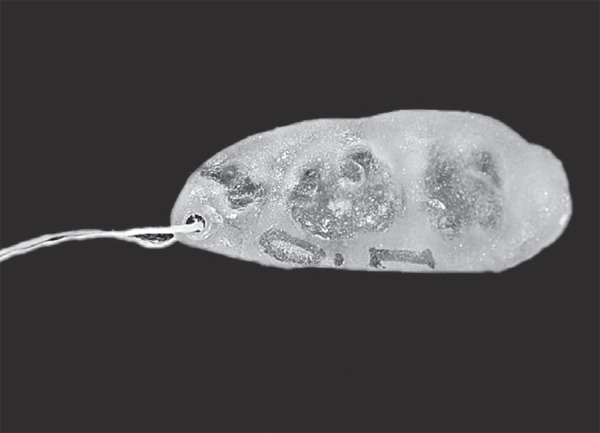
Acrylic resin device used for occlusal fixation in CR

A 36-year and 9 month-old female, Caucasian patient sought orthodontic treatment with a chief complaint of anterior crossbite and mandibular prognathism. Her clinical examinations revealed a great difference between maximal intercuspation (MI) and centric relation (CR) in the anteroposterior and vertical direction, with a major impact on face and occlusion (Fig 1). With a view to performing a safe morphological analysis, an acrylic interocclusal device was manufactured (Fig 2) with the mandible in CR, given that this position favored qualitative analysis and, as a result, improved prognosis for a compensatory treatment. For this reason, two analyses were carried out in order to obtain patient's facial morphological diagnosis: one in MI, and another in CR. Her frontal facial analysis in MI revealed little asymmetry, chin deviated to the right, severe anterior proclination, good zygomatic projection, compressive labial seal and decreased lower third. Nevertheless, in CR, analysis revealed that vertical shortening and facial asymmetry were minimized, and a more balanced face without signs of chin deviation (Figs 1A, 1B). Profile analysis confirmed the aforementioned characteristics, both in MI and CR, as well as an increased chin-neck line in MI. Nasolabial angle was closed partially due to the compensation of maxillary incisors, but, especially in MI, due to forced labial seal and consequent decreased ALFH (Figs 1C, 1D). Smile analysis revealed good incisors exposure with normal inclination and slight deviation of the occlusal plane, which was later justified by unilateral crossbite on the right side (Fig 1E).

Occlusal assessment in MI revealed a sagittal relationship between maxilla and mandible of 3/4 of Class III on the right side and 1/4 on the left side, with anterior and posterior crossbite on the right side without involving second molars. Mandibular incisors were retroclined at a clearly compensatory position as a result of a decreased maxillomandibular step. Mid lines coincided with the facial midline (Fig 1F, 1K).

**Figure 3:**
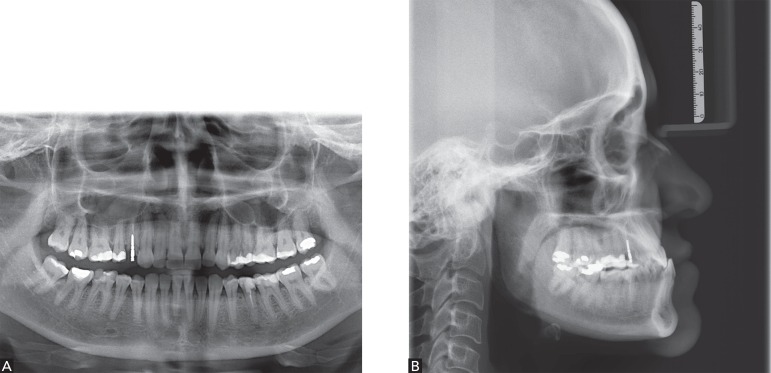
Initial photographs: **A**) Panoramic radiograph; **B**) Profile radiograph

A panamoramic radiograph confirmed the presence of all permanent teeth, with third molars in occlusion and a periodontal condition that was consistent with patient's age. Tooth #14 had a provisional crown as well as an intracanal post and presented favorable conditions for orthodontic treatment onset (Fig 3A).

From a skeletal standpoint, morphological evaluation of the cephalogram revealed a negative maxillomandibular step with mild mandibular prognathism, especially due to an anticlockwise mandibular rotation, given that the cephalogram was taken at maximal intercuspation. Although mandibular incisors were lingually tipped and strongly compensated, they were also well inserted into the symphysis. Conversely, maxillary incisors were well positioned in the maxillary bone (Fig 3B).

After collecting all necessary occlusal, functional, cephalometric and face-morphology examinations, and evincing a deviation from CR to MI, we came up with the following diagnosis: adult patient, mild skeletal Class III, brachyfacial, borderline for Short Face and with an acceptable facial pattern. Class III relationship on the right side and 1/4 on the left side, with anterior and posterior crossbite on the right side. Well-positioned maxillary incisors and retroclined mandibular incisors in relation to the bone. Patient's morphological analysis of the face in CR (Figs 1B, 1D) reinforced the need for compensatory treatment that aimed at increasing volume in the maxillary arch and restricting the mandibular arch. The absence of crowding in the mandibular arch favored such treatment goal, although it hindered an increase in circumference in the maxillary arch.

**Figure 4:**
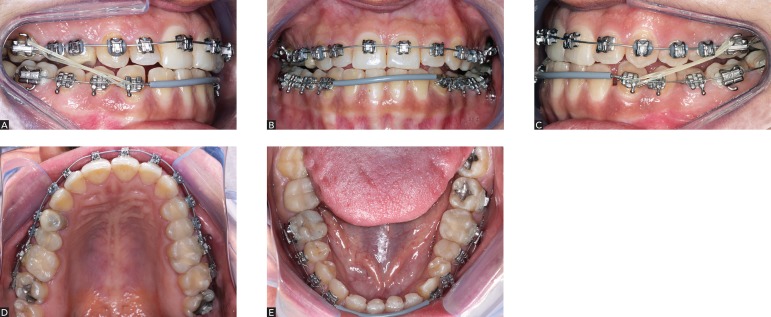
Intraoral photographs at the beginning of complete levelling in the maxillary arch and partial levelling in the mandibular arch.

Treatment plan included the use of Damon MX^®^ standard self-ligating brackets (Ormco), with torque of +12ºapplied to central incisors, +8ºto lateral incisors and 0ºto canines, respecting the need for increasing volume in the maxillary arch within esthetic limits - which could be exceeded with the use of high torque brackets (+17°, +12° and +6°, respectively) or by producing, by means of low torque brackets (+7° and +3°, for central and lateral incisors), a weaker protrusion, insufficient to correct crossbite. The mandibular arch received Capelozza^®^ Prescription III brackets (Abzil, 3M(tm)). At first, mandibular incisors were not included in order to avoid protrusion (given that anterior lower crowding was quite discrete) which could have been produced by initial and random leveling of lower teeth (Fig 4).

Capelozza^®^ Prescription III brackets (Abzil, 3M(tm)) have a very positive characteristic that favors the therapeutic goal recommended for this patient: maximum preservation of the mandibular arch or, in small proportions, a modest increase in the natural compensatory characteristics. Torque of -6ºwould be applied to mandibular incisors to this end. In other words, mandibular incisors would be severely lingually tipped by the mechanics to which brackets would contribute. Although there was no intention of further using rectangular wires in the mandibular arch, this procedure does not break with the concept of maintaining the original form of the arch. Without a doubt, the key factor to achieve treatment success in this compensatory game is the absolute economy of angulations provided by brackets with no angulation bonded from canine to canine, which results in little protrusion and requires less space during leveling.

In this approach, which the orthodontist assumes total control of treatment, bracket bonding was individualized and maxillary incisors were more cervically bonded so as to adjust the incisal curvature of final smile and, at the same time, allow low reading of strong torque embedded in maxillary brackets. Before interpreting this as nonsense, one should remember that, in this case, treatment approach intended to increase maxillary protrusion in accordance with esthetic limitations. Additionally, there is speculation that this treatment protocol stimulates greater bodily buccal movement. Should mandibular incisors be bonded, they were more cervically positioned in relation to the vestibular axis point with the height of previously leveled canines as reference. All aforementioned alterations are favorable in compensatory cases of skeletal and dental Class III, as they favor good overbite as well as functional anterior guidance.

With a view to enhancing the position of mandibular orthopedic stability and deconstructing maximal intercuspation, fixed stops made of composite resin were bonded to the lingual surface and incisal third of mandibular incisors with balanced and uniform occlusal contact with antagonist teeth (Fig 4E). This measure favors buccal movement of teeth involved in crossbite, stimulates extrusion of posterior teeth within the posterior interocclusal space created to produce gain in vertical dimension of occlusion (VDO), and, at the same time, improves treatment mechanical efficacy by producing an effect of occlusal unlocking.

**Figure 5:**
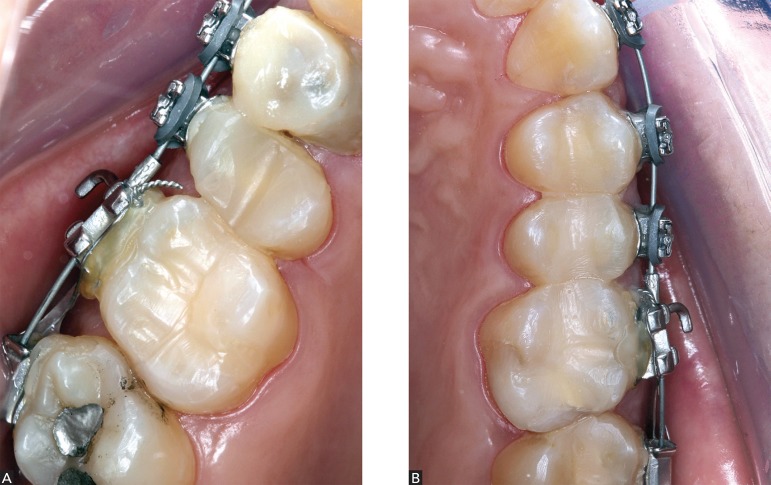
Photographs depicting right and left maxillary quadrants, highlighting stops placement and the use of elastic ligatures.

Also, with a view to directing movement towards the areas of interest, which were carefully investigated, stops were bonded to the mesial surface of first molars and the arch was adjusted with a space of 2 mm between the wire and the bracket. In other words: The wire was mesially fitted on first molars and passed 2 mm away from the maxillary incisors, thereby stimulating protrusion of these teeth. Additionally, elastomers were placed on premolars and canines so as to concentrate the expansion in the anterior region, thereby meeting the primary treatment objective (Fig 5). After correcting anterior crossbite, mechanics was directed towards teeth #14 and 15.

**Figure 6:**
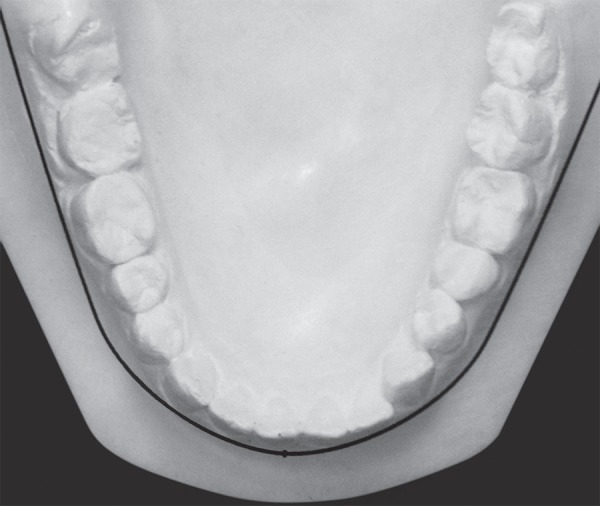
C6A7 objective anatomic individual diagram

With a view to maintaining the compensatory characteristics, which are also related to the form of the arch, C6A7 diagram was chosen (Fig 6) for favoring slight retroclination of mandibular incisors and protrusion of maxillary incisors. Additionally, in the posterior region, it respected the mandatory dentoalveolar compensatory mechanism of the mandibular arch.^16^

Two months after the onset of leveling in the maxillary arch, a mandibular appliance was installed with immediate use of Class III 5/16-in rubber bands supported by hooks placed on maxillary first molars and mandibular canines. This measure immediately prevented mandibular protrusion and, at the same time, produced space gain necessary for future leveling of mandibular incisors without stimulating buccal inclination.

**Figure 7:**
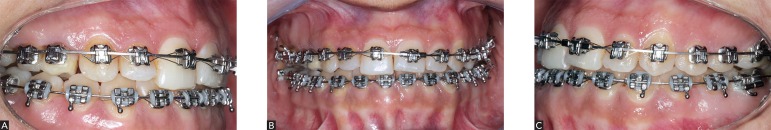
Intraoral photographs at the end of leveling in the maxillary arch (with 0.019 x 0.025-in wire) and in the mandibular arch (with 0.018" steel wire

**Figure 8:**
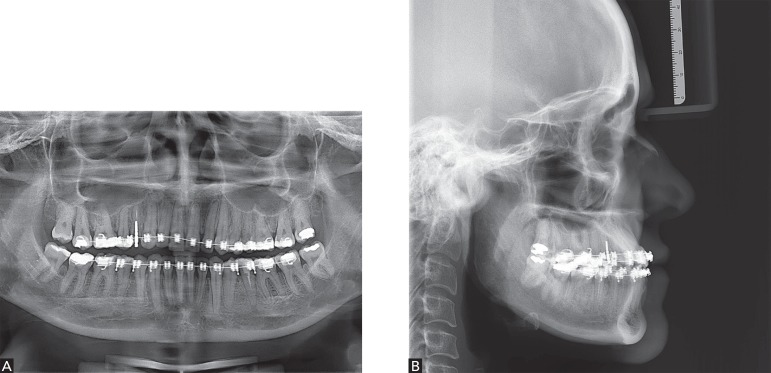
Radiographs at treatment completion: **A**) Panoramic; **B**) Profile radiograph.

Without a doubt, this treatment phase was the most difficult in terms of mechanics, given that any careless procedure could worsen anteroposterior relationship between the maxilla and the mandible and, as a result, create greater demand for treatment of potential side effects. From this initial phase on, maxillary leveling was conducted with a sequence of archwires evolving to 0.019 x 0.015-in steel wire. As for the mandibular arch, the sequence of archwires stopped at 0.018 steel wires because rectangular wires were not necessary for additional angulation or inclination reading. During the formatting phase of the mandibular arch, morphology was consistent with the initial treatment goals. Moreover, even if individualized brackets were used, they were not completely customized and, for this reason, their maximum expression may not suit this type of patient (Fig 7). In the final treatment phase, panoramic and lateral radiographs were taken with a view to assessing tooth positioning and potential biological costs inherent to orthodontic treatment (Fig 8).

**Figure 9:**
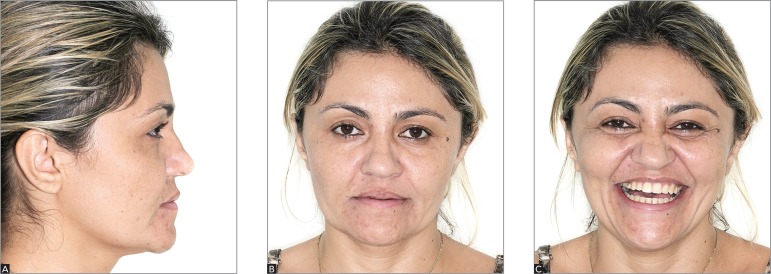
Final extraoral photographs.

**Figure 10:**
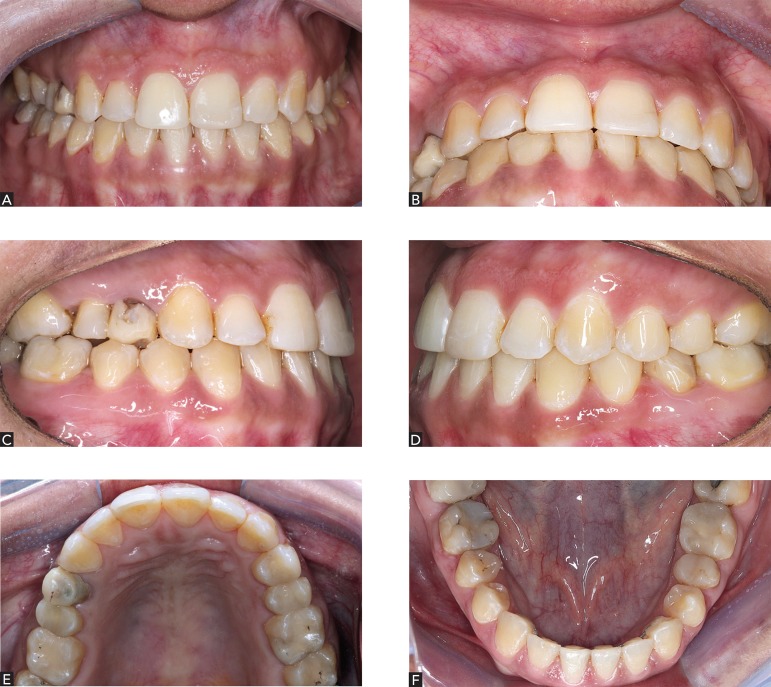
Final intraoral photographs.

Figure 9 shows slight, yet major improvements in lip contact. It also depicts decreased asymmetry initially shown at maximal intercuspation in frontal view. Figure 10 shows good occlusal relationship achieved after removing the appliance.

**Figure 11:**
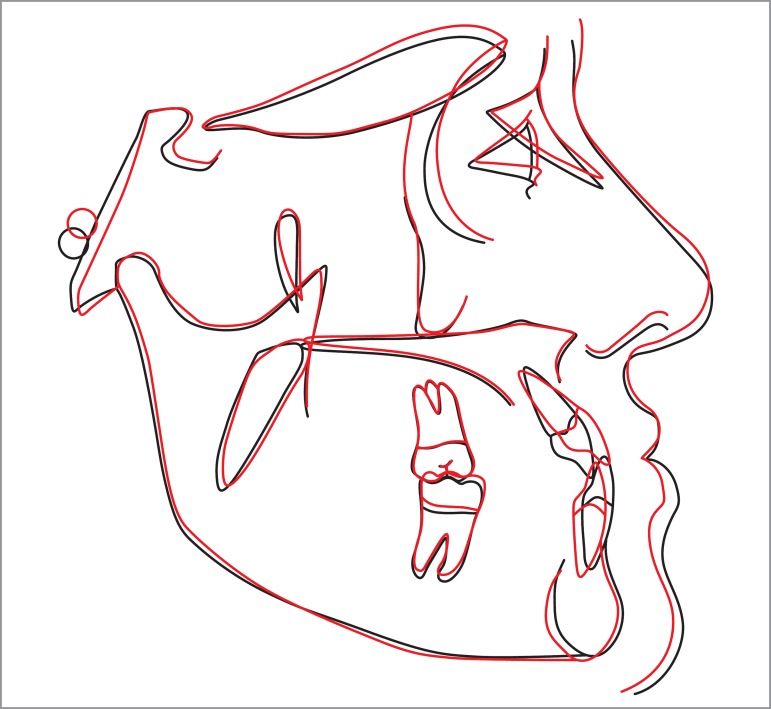
Initial (black) and final (red) cephalometric tracings superimposition.

Figure 11 shows initial and final cephalometric tracings superimposition at treatment completion, which allowed an accurate analysis of the mechanisms that enabled occlusal adjustment. Improvements were achieved due to a set of several small adjustments, namely: correction of discrepancy between CR and MI, retroclination of mandibular incisors and protrusion of maxillary incisors. All these factors added up to magnify the positive impacts on patient's occlusion and face as well as to allow transverse expansion of the maxillary arch and crossbite correction.

Treatment lasted for 15 months, with a total number of 10 visits since the appliance was firstly installed in the maxillary arch until it was removed.

## CASE REPORT 2

**Figure 12:**
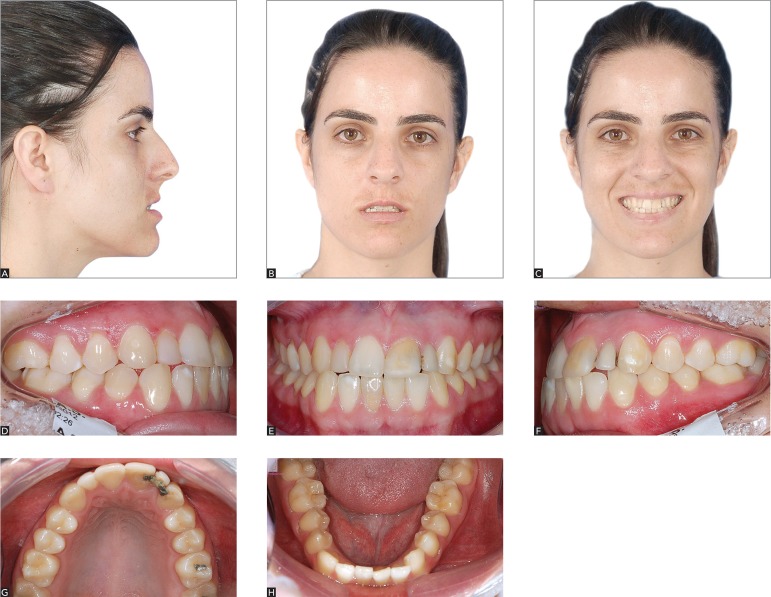
Initial extra and intraoral photographs.

A 26-year and 2-month-old female, Caucasian patient sought orthodontic treatment with chief complaint of lack of space for implant placement at tooth #22 site and small-sized tooth #12. Her profile analysis revealed maxillary deficiency and unsatisfactory lip contact with her lower lip ahead her upper lip and open nasolabial angle (Fig 24A). Her frontal facial analysis revealed a balanced face with good acceptability (Fig 12 B). Her smile was characterized by lack of space, disproportional maxillary lateral teeth and tooth #21 darkened by endodontic treatment (Fig 12C).

Her occlusal analysis revealed Class III subdivision malocclusion of 1/4 on the right side, crossbite on tooth #12 as well as decreased overbite and overjet. Her mandibular arch showed evident compensation, with retroclined incisors and mandibullar canines with no mesial angulation. Mid lines coincided with the facial midline (Fig 12D, 12H).

**Figure 13:**
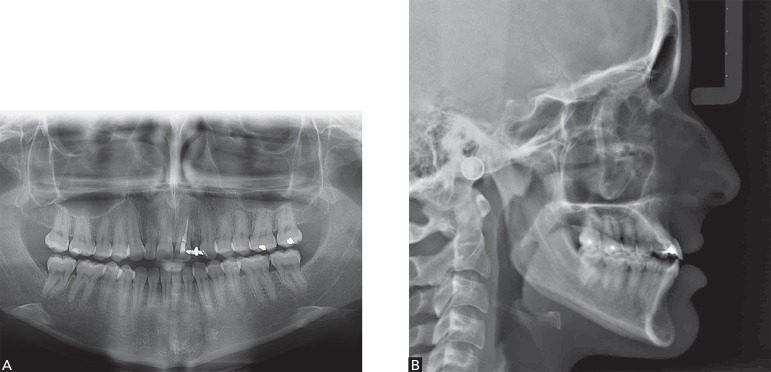
radiographs: A) Panoramic raA B diograph; B) Profile radiograph.

Panamoramic radiograph confirmed maxillary and mandibular third molars as well as tooth #22 agenesis corrected by an adhesive prosthesis bonded to teeth #21 and 23. She presented general periodontal condition that favored orthodontic treatment (Fig 13A).

From a skeletal standpoint, the cephalogram revealed a negative maxillomandibular step with mild maxillary deficiency and differences between palatal and mandibular planes. Maxillary incisors were buccally tipped as expected. However, mandibular incisors counteracted occlusal analysis as they were well positioned in the symphysis (Fig 13B).

Diagnosis was as follows: adult patient, mild skeletal Class III, dolichofacial with acceptable facial pattern, especially from frontal view. Class III relationship on the right side, with anterior crossbite on tooth #12, decreased overbite and overjet, agenesis of tooth #22 and increased buccal tipping of maxillary incisors.

Patient's self-perception of facial normality in frontal view reinforced the need for compensatory treatment while eliminating the need for absolute corrective treatment by means of orthognathic surgery for maxillary advancement. In this context, treatment plan was directed towards the protocol presented herein: the use of Damon MX^®^ (Ormco) self-ligating brackets. Unlike case 1, high torque prescription was chosen for the maxillary arch (CI +17 º, IL +10º, C +7º) as it required greater protrusion and expansion, both of which were justified by more expressive buccal torque applied to the maxillary arch and Capelozza® Prescription III brackets (Abzil, 3M^TM^) used in the mandibular arch.

In case 2, the greatest challenge was to increase overbite and overjet while opening spaces for appropriate rehabilitation of maxillary lateral incisors without producing the effect of reversing the incisal curvature at smiling - which is quite common in cases requiring major compesantion of maxillary incisors. In order to control such effect, Prescription III brackets were used in the mandibular arch with brackets more cervically bonded on maxillary lateral and central incisors.

**Figure 14:**
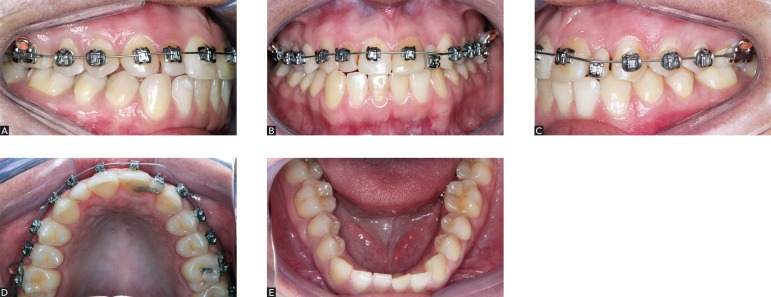
Initial treatment approach with leveling of maxillary arch performed with stops placed on the mesial surface of molars, elastic ligatures on anchorage teeth and the use of 0.014-in heatactivated with anterior slack.

Once again, with a view to directing movement towards the areas of interest, stops were bonded to the mesial surface of first molars, thereby producing a space between the 0.14" heat-activated wire and the bracket in the anterior region of the maxillary arch. Additionally, elastic ligatures were used from right and left premolars to right canine as - transversely speaking - those teeth functioned as reference of normality. Treatment onset on the maxillary arch was of paramount importance, and so was installing the appliance on the mandibular arch 40 days after using 0.014 x 0.025-in heat-activated wire in the maxillary arch - 4 months after treatment onset. In other words, it was installed after the maxillary arch form was established, which used to be limited, and was now more expanded and defined. In order to favor greater anterior overbite, maxillary second molars were not included in leveling. This set of actions is definitively in accordance with the therapeutic goals previously established for patient's compensatory treatment (Fig 14).

**Figure 15:**
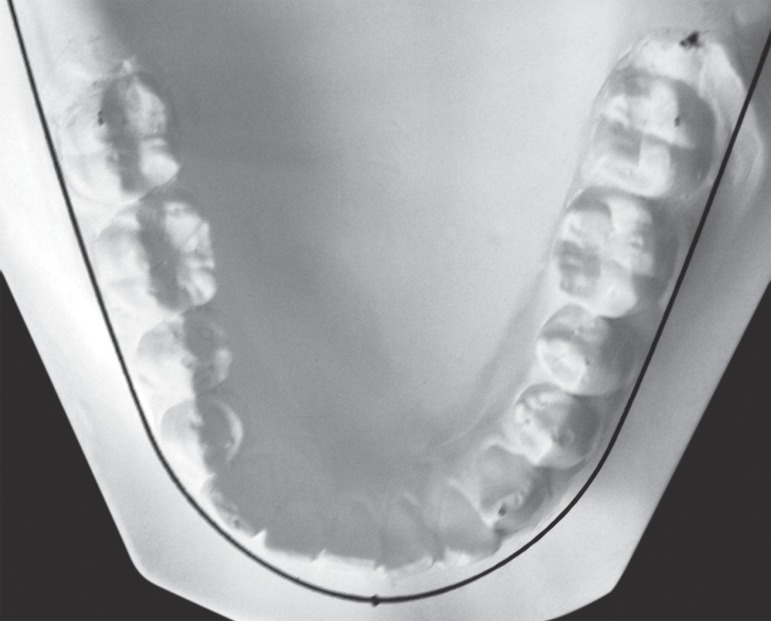
C4A7 objective anatomic individual diagram.

C4A7 diagram was chosen (Fig 15) for providing greater freedom to improve the form of the mandibular arch, which was allowed by the great demand for space in the maxillary arch.

The sequence of wires used in the maxillary arch was as follows: 0.014" heat-activated; 0.014 x 0.025-in heat-activated; 0.017 x 0.025-in TMA; 0.019 x 0.025-in TMA and 0.019 x 0.025-in steel wire. As for the mandibular arch, 0.014 NiTi superelastic and 0.016 NiTi superelastic wires were mesially fitted, followed by 0.018" steel wire installed with omega loops.

**Figure 16:**
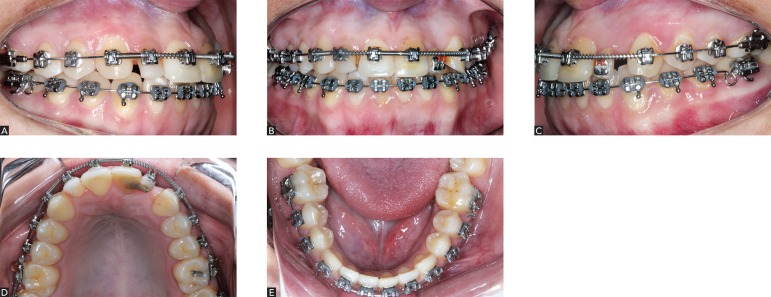
Final leveling phase with open and closed springs installed to increase in circumference in the region of teeth #12 and #22.

Figure 16 shows the effect produced with the use of open and closed springs to equalize the space necessary for proper rehabilitation of teeth #12 and #22.

**Figure 17:**
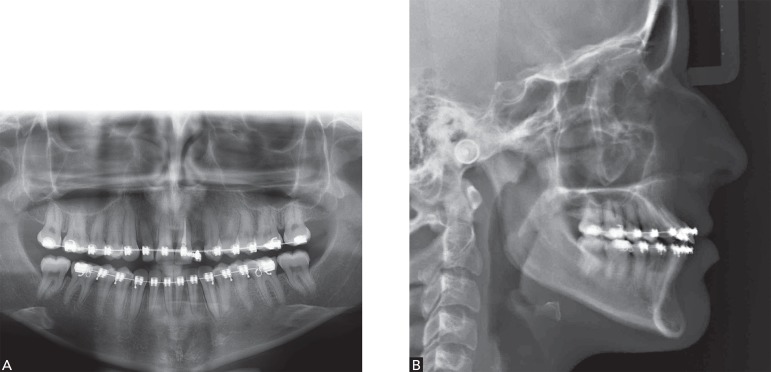
Radiographs at treatment compleA B tion: **A**) Panoramic; **B**) Profile radiograph.

Final panoramic and profile radiographs not only certify safe and trustful results, but also confirm intraosseous space gain for future implant placement on tooth #22 site (Fig 17).

**Figure 18:**
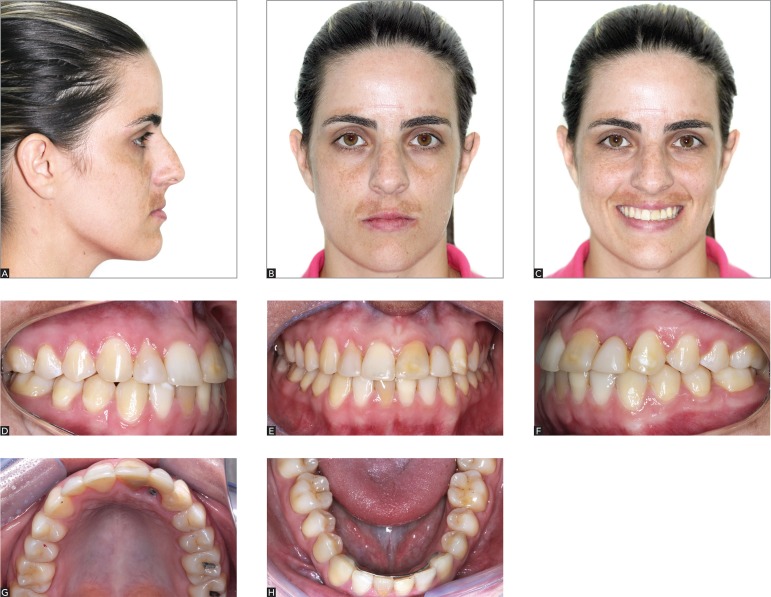
Final extra and intraoral photographs.

Treatment produced considerable improvements and discreet, yet extremely positive benefits for the face. Thus, it proves the protocol adopted herein to be efficient with regard to the therapeutic goals previously established (Fig 18).

**Figure 19:**
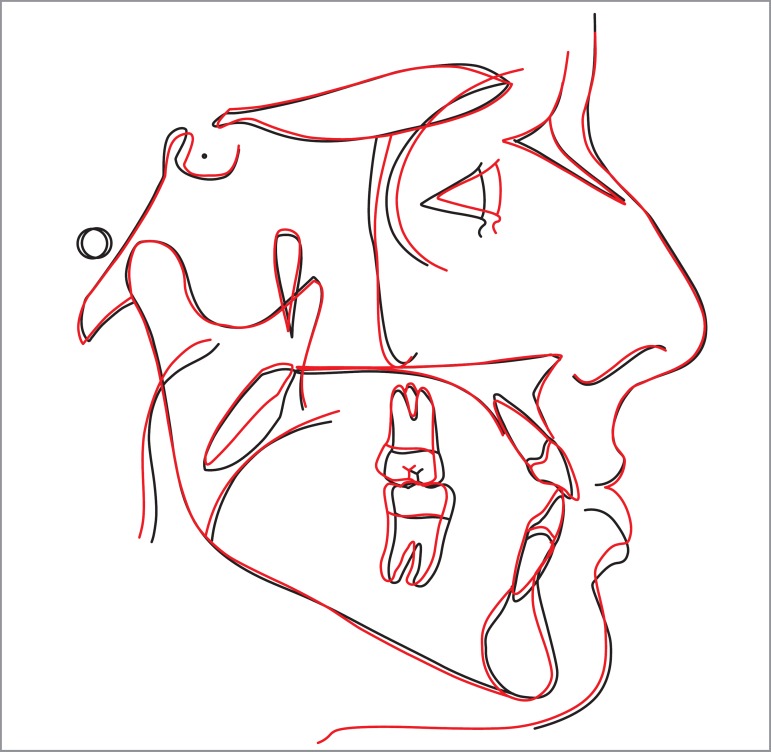
Initial (black) and final (red) cephalometric tracings superimposition

Cephalometric tracings superimposition helps us understand that right choices were made with a view to achieving functional and esthetic balance of a malocclusion that presents compensatory characteristics inherent to both skeletal Class III and sagittal relationship between maxilla and mandible aggravated by agenesis in the anterior region of the maxillary arch (Fig 19).

Treatment lasted for 18 months, with a total number of 11 visits.

## CASE REPORT 3

**Figure 20:**
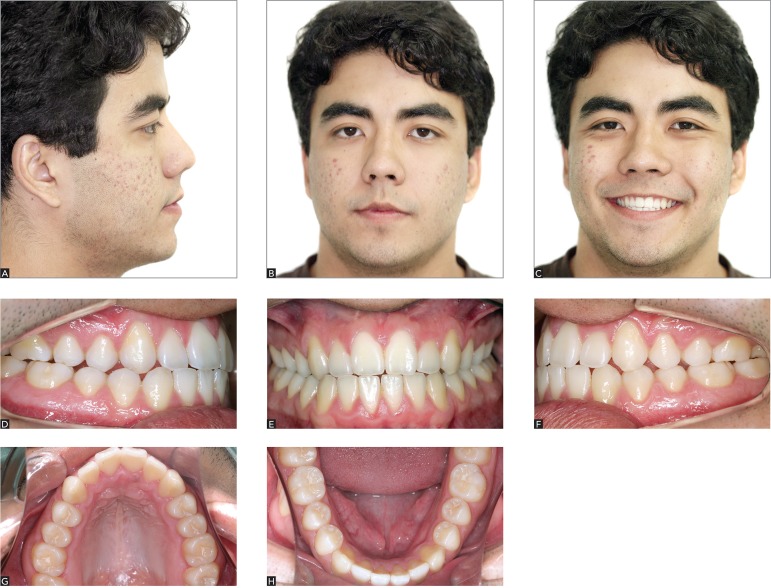
Initial extra and intraoral photographs.

A 23-year-old male, Japanese-descendent patient sought orthodontic treatment with chief complaint of unilateral crossbite on the right side and mandibular shift to the right. His frontal analysis revealed vertically balanced face with discreet laterognathism. His profile analysis revealed clearly balanced maxillomandibular relationship with passive lip seal, slightly open nasolabial angle, well-defined mentolabial sulcus and normal chin-neck line. At similing, the patient presented some alterations such as reversed incisal curvature in relation to the lower lip curve, asymmetry in the positioning of teeth #13 and 23, and increased lingual inclination of teeth #14 and 15 (Figs 20A, B, C). His occlusal analysis (Figs 20D to 20H) revealed bilateral Class III sagittal relationship more severe in the second premolar region than in the canine region. This difference in magnitude may be explained by the level of compensation present in mandibular canines and premolars that resigned their mesial angulations. Transverse relationship was impaired by unilateral crossbite on the right side and decreased overbite as well as overjet in the anterior region. Frontal intraoral view revealed a 2-mm deviation of the lower midline to the right coinciding with mandibular skeletal deviation.

**Figure 21:**
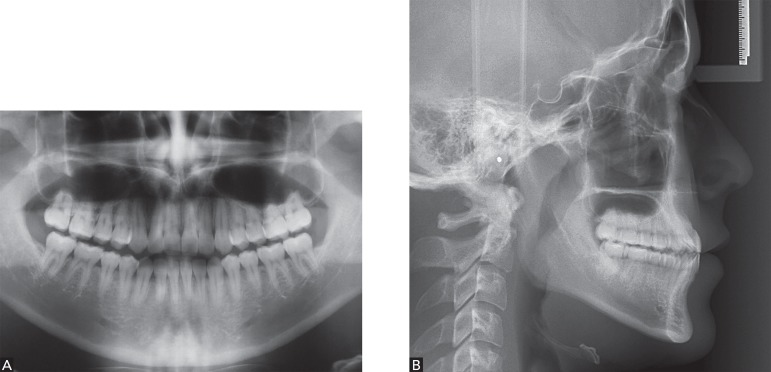
Initial radiographs: A) Panoramic radiograph; B) Profile radiograph.

Panamoramic radiograph revealed periodontal and structural health that favored orthodontic treatment. Third molars had been previously extracted (Fig 21A). Morphological exams of the cephalogram confirmed all aforementioned positive facial characteristics and revealed something new: vertical maxillary excess unable to negatively affect patient's face or smile. Maxillary and mandibular incisors were well positioned into the jaws (Fig 21B).

Unlike the aforementioned patients, this patient was diagnosed as skeletal Class I with mild laterognathism to the right, dolichofacial and pleasant face. Bilateral Class III relationship with unilateral crossbite on the right side and absence of overbite and overjet. He was asked about the possibility of undergoing orthognathic surgery, since the procedure would be the only one capable of correcting asymmetry - one of his chief complaints. Nevertheless, given that mild mandibular shift did not worsen after a year, the possibility of surgery was discarded and compensatory treatment was chosen to solve patient's occlusal problems, thereby enduring his small skeletal defect. Although the patient was skeletal Class I, the relationship between maxilla and mandible was Class III and granted him occlusal characteristics of Class III. For this reason, he was treated under the same protocol employed in cases 1 and 2.

**Figure 22:**
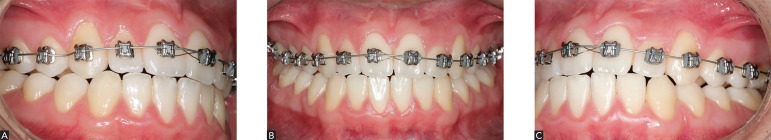
**A**) Initial treatment approach with leveling of maxillary arch performed with stops placed on the mesial surface of molars, elastic ligatures on anchorage teeth and the use of 0.014" heat-activated with anterior space between teeth #11 and #14.

Once again, we faced the need for controlled maxillary protrusion and expansion as well as restriction of both in the mandibular arch. Figure 22 shows similarities with the aforementioned protocol: treatment onset on the maxillary arch, use of stops and elastic ligatures with a view to achieving protrusion and expansion in the right anterior and lateral region.

**Figure 23:**
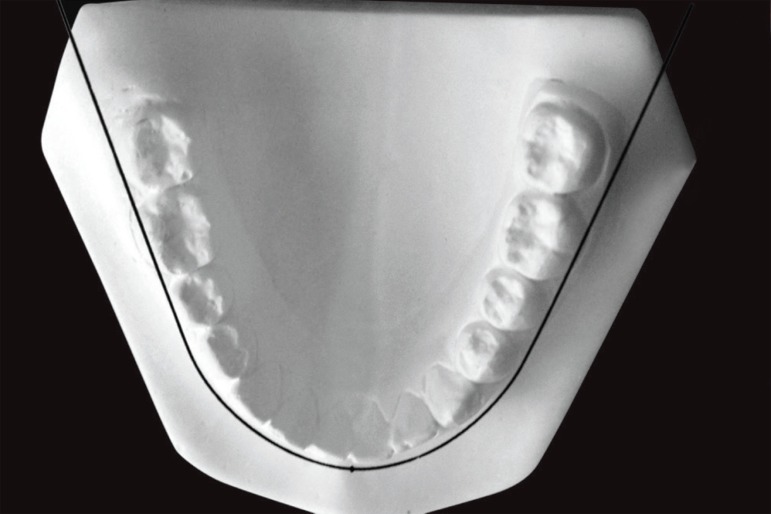
C5A9 objective anatomic individual diagram.

C5A9 diagram (Fig 23) was chosen to preserve the form of the mandibular arch, given that unilateral crossbite on the right side, without deviation from MI to CR, was a predictive factor of potential difficulties in achieving proper transverse control - especially in the case of an adult patient.

**Figure 24:**
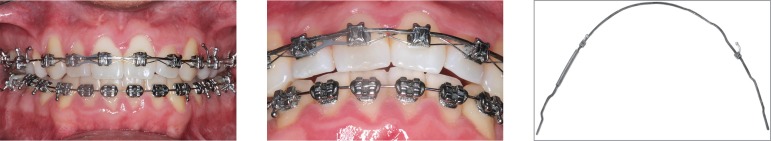
Final leveling phase with individualized bends for treatment finishing: buccal steps on teeth #16, 26, 12 and 13; and "Z" bend on tooth #21.

The sequence of wires used in the maxillary arch was as follows: 0.014-in; 0.016-in; 0.014 x 0.025-in; 0.018 x 0.025-in heat-activated; 0.019 x 0.025-in TMA and 0.019 x 0.025-in steel wire. As for the mandibular arch, the following arches were used: 0.014" NiTi superelastic wire mesially fitted; 0.016 and 0.018-in steel wires with omega loops. This patient required compensatory bends (Fig 24) for a more individualized treatment finishing, especially due to laterognathism.

**Figure 25:**
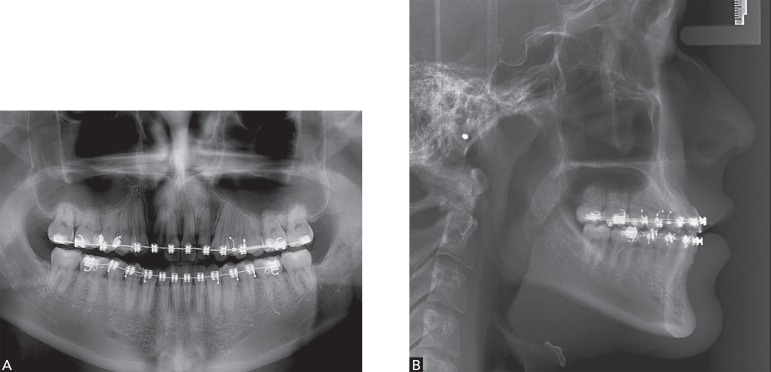
Radiographs at treatment completion: A) Panoramic radiograph, B) CephalometA B ric cephalogram.

**Figure 26:**
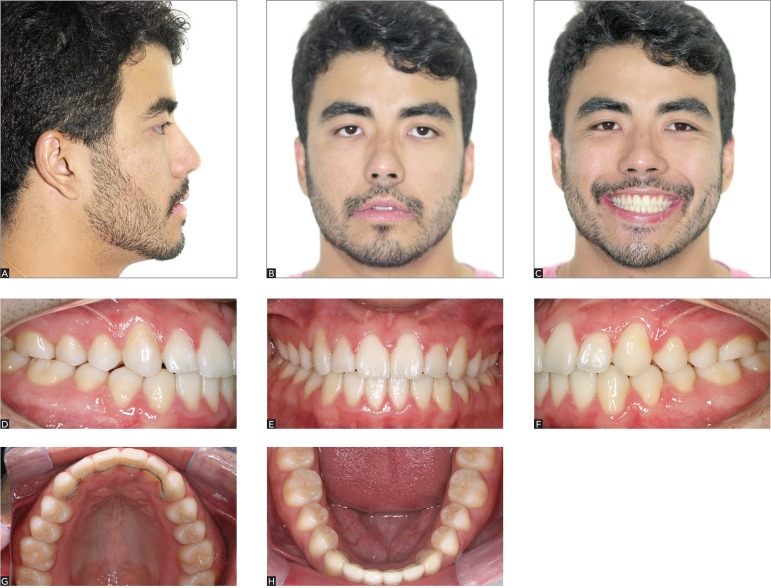
Final extra and intraoral photographs.

Final panoramic and lateral radiographs reveal absolute control (Fig 25). Figure 26 certifies protocol efficacy. Patient's face did not change, as expected. Nevertheless, gains in overbite and overjet, correction of unilateral crossbite and Class I relationship established between canines provided him with a functional routine and expressive esthetic benefits.

**Figure 27:**
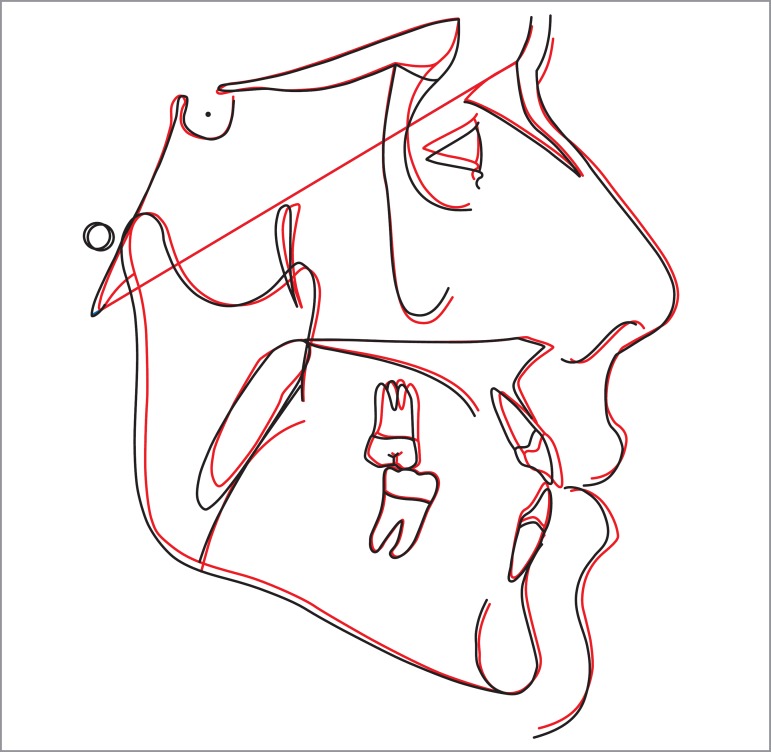
Initial (black) and final (red) cephalometric tracings superimposition.

Initial and final cephalometric tracings superimposition reveals that the effects described in case report 1 and 2 were repeated in case 3 (Fig 27).

Treatment lasted for 24 months, with a total number of 17 visits. Treatment time was greater than expected due to unilateral crossbite, the need for individualization bends and the clinician's learning curve - since this was the first patient treated under the protocol described herein.

## FINAL CONSIDERATIONS

The gains in efficiency of alignment and leveling produced by self-ligating brackets have not been scientifically proved. Some recent studies do not seem favorable to confirm the greater productivity of this system, since most of them aim at comparing the magnitude of movement during alignment and leveling without considering the individual variations of the samples.^10,17 ^From this point of view, self-ligating brackets would be just a more practical method employed to fit and remove archwires. Nevertheless, directing mechanics associated with bracket individualization towards flexile therapeutic goals seems to enhance treatment outcomes. Carefully using stops and elastic ligatures to manage friction in self-ligating bracket systems used in areas where movement is less required is a good example of how to explore the maximum productivity of this system, and justifies the methodology employed to treat the patients reported herein.

Using individualized Capelozza^®^ Prescription III brackets (Abzil, 3M(tm)) in the mandibular arch to treat Class III is essential to yield more esthetically tolerated results, given that maximum maintenance of the arch form creates possibilities of moderate gains in the maxillary arch without hindering smile esthetics. This occurs because the ideal morphology for sagittal correction of the arches is limited by smile reading; thereby giving the orthodontist the opportunity to create a less protrusive and less expansive maxillary arch than he would mechanically do.

It seems imperative to treat these malocclusions by means of absolutely individualized methods, seeking to preserve what should remain and strictly change what must be corrected. Treatment that starts on a reasonable or poor occlusal morphology should continuously evolve to improvements so as to prevent a greater demand for treatment.
